# miR-142-3p encapsulated in T lymphocyte-derived tissue small extracellular vesicles induces Treg function defect and thyrocyte destruction in Hashimoto’s thyroiditis

**DOI:** 10.1186/s12916-023-02914-7

**Published:** 2023-06-06

**Authors:** Genpeng Li, Linye He, Jing Huang, Jiaye Liu, Wenjie Chen, Jinjing Zhong, Tao Wei, Zhihui Li, Jingqiang Zhu, Jianyong Lei

**Affiliations:** 1 Division of Thyroid Surgery, Department of General Surgery, West China Hospital, Sichuan University, Chengdu, 610041 China; 2 The Laboratory of Thyroid and Parathyroid Disease, Frontiers Science Center for Disease-Related Molecular Network, West China Hospital, Sichuan University, Chengdu, 610041 China; 3 Department of Pathology, West China Hospital, Sichuan University, Chengdu, 610041 China

**Keywords:** Hashimoto’s thyroiditis, MicroRNAs, Small extracellular vesicles, T lymphocytes

## Abstract

**Background:**

Hashimoto’s thyroiditis (HT) is an organ-specific autoimmune disease characterized by lymphocyte infiltration that destroys thyrocyte cells. The aim of the present study was to elucidate the role and mechanisms of tissue small extracellular vesicle (sEV) microRNAs (miRNAs) in the pathogenesis of HT.

**Methods:**

Differentially expressed tissue sEV miRNAs were identified between HT tissue and normal tissue by RNA sequencing in the testing set (*n* = 20). Subsequently, using quantitative real-time polymerase chain reaction (qRT‒PCR) assays and logistic regression analysis in the validation set (*n* = 60), the most relevant tissue sEV miRNAs to HT were verified. The parental and recipient cells of that tissue sEV miRNA were then explored. In vitro and in vivo experiments were further performed to elucidate the function and potential mechanisms of sEV miRNAs that contribute to the development of HT.

**Results:**

We identified that miR-142-3p encapsulated in T lymphocyte-derived tissue sEVs can induce Treg function defect and thyrocyte destruction through an intact response loop. Inactivation of miR-142-3p can effectively protect non-obese diabetic (NOD).H-2^h4^ mice from HT development display reduced lymphocyte infiltration, lower antibody titers, and higher Treg cells. Looking at the mechanisms underlying sEV action on thyrocyte destruction, we found that the strong deleterious effect mediated by tissue sEV miR-142-3p is due to its ability to block the activation of the ERK1/2 signaling pathway by downregulating *RAC1*.

**Conclusions:**

Our findings highlight the fact that tissue sEV-mediated miR-142-3p transfer can serve as a communication mode between T lymphocytes and thyrocyte cells in HT, favoring the progression of HT.

**Supplementary Information:**

The online version contains supplementary material available at 10.1186/s12916-023-02914-7.

## Background

Hashimoto’s thyroiditis (HT), an organ-specific autoimmune disease with incidence of 0.3–1.5/1000 in population, is characterized by lymphocyte infiltration, which results in destruction of the thyroid follicular structure and leads to hypothyroidism in approximately 20–30% of patients [1, 2]. Patients suffering from hypothyroidism may require lifelong oral administration of a synthetic hormone, the levo-thyroxine 4 (L-T4), to maintain normal hormone levels [3, 4]. Understanding the dialogue between infiltrating lymphocytes and thyrocyte cells is crucial to promote the design of novel strategies to prevent the progression of this disease. Part of the detrimental effects of the lymphocytes on thyrocyte cell survival is known to be exerted through the release of autoantibodies and pro-inflammatory cytokines [1, 5–7]. Autoantibodies are able to induce the antibody-dependent cellular cytotoxicity, and the complement-dependent cytotoxicity, contributing to the thyrocyte death and atrophy [6]. Pro-inflammatory cytokines such as interferon-gamma (IFNγ) can induce the secretion of CXCL9/10/11 in primary granulosa cells, and further promote the migration of T lymphocytes into the follicle by binding to CXCR3, thereby perpetuating an inflammatory cascade in the follicular microenvironment [7]. Recently, the strong deleterious effect of lymphocyte-derived small extracellular vesicles (sEVs) on parenchyma cells in autoimmune diseases has been proposed [8, 9], which provides a new perspective for interpreting the pathogenesis of autoimmune diseases.

sEVs are released by a variety of cells and are presented in cell culture supernatants, body fluids and tissues [10]. Compared to sEVs obtained from supernatants and body fluids, tissue sEVs, existence in the interstitial space, possess advantages of authenticity, purity and specificity [10, 11]. Tissue sEVs can contain a wide range of components from their parental cells, including lipids, proteins, and coding and noncoding RNAs, which can be transferred to recipient cells to affect biological activity, such as tumor metastasis and inflammatory activation [8–10, 12].

MicroRNAs (miRNAs), more abundant and powerful than other “cargos” in sEVs [10, 13], can regulate target gene expression [14]. Dysregulation of miRNA transcription and function has been associated with a variety of autoimmune diseases [15, 16]. The previous report identified a five-signature miRNA that could be an independent risk factor for developing autoimmune thyroid diseases [17]. In addition, a recent study determined that altered expression of miR-22-5p and miR-142-3p was associated with higher levels of thyroid antibodies, suggesting their contribution to the pathogenesis of HT [18]. However, those studies mainly focus on circulating miRNAs which cannot well reflect the state of the microenvironment in HT due to the lack of tissue specificity.

Here, we aimed to investigate the role and potential mechanisms of tissue sEV miRNAs in the pathogenesis of HT. We identified that upregulated tissue sEV miR-142-3p, derived from T lymphocytes, can be internalized by thyrocyte cells in HT. The biological effects of miR-142-3p on parental and recipient cells and the mechanisms by which miR-142-3p promotes the development of HT were further reported. Taken together, our results suggest that the delivery of miR-142-3p mediated by tissue sEVs released by T lymphocytes could be part of the mechanisms leading to HT progression.

## Methods

### Study subjects and cell line

This study was approved by the institutional ethics review board of West China Hospital of Sichuan University (Number: 2020 (4)), and informed consent was obtained from each patient. Eighty patients with unilateral thyroid neoplasms requiring total thyroidectomy were included and divided into two independent sets, including twenty patients in the testing set and sixty patients in the validation set. The comparisons indicated that patients in each set had similar demographics and clinical characteristics (Additional file 1: Table S1). Thyroid-stimulating hormone (TSH), free thyroxine 3 (FT3), free thyroxine 4 (FT4), and levels of thyroglobulin antibody (TgAb) and thyroperoxidase antibody (TPOAb) were measured at the time of surgery. HT was diagnosed by thyroid histopathological examination characterized by diffuse lymphocytic infiltration and destruction of thyroid follicle cells or combined with elevated TgAb and TPOAb [1]. Thyroid tissue from the contralateral lobe of the neoplasms was collected and stored at − 80 °C before use.

The human thyrocyte cell line Nthy-ori-3–1 was purchased from EK-Bioscience Biotechnology Co., Ltd. (Shanghai, China) and cultured in RPMI-1640 (Invitrogen). The media were supplemented with 10% fetal bovine serum and 1% penicillin/streptomycin (Invitrogen), and the cells were grown at 5% CO_2_ and 37 °C.

Human CD4^+^ T lymphocytes were isolated from thyroid tissue and peripheral venous blood by using fluorescence-activated cell sorting and the EasySep™ Human T-cell Enrichment Kit (Stemcell), respectively. To ensure the purity of T-cell populations, they were characterized by flow cytometry with FITC anti-human CD3 (BioLegend) and APC anti-human CD4 (BioLegend) antibodies. For further staining of Foxp3, cells were first fixed and permeabilized using a commercial cell fixation/permeabilization kit and then incubated with phycoerythrin (PE)-labeled anti-Foxp3 antibody (Biolegend). For Treg isolation, a Treg isolation kit (Miltenyi Biotec) was used to purify Tregs from approximately 4 × 10^7^ human CD4^+^ T lymphocytes isolated from peripheral blood according to the manufacturer’s instructions. Approximately 2 × 10^6^ Treg cells were obtained and cultured at a concentration of 0.5 × 10^6^ cells/mL in a 6-well plate. The isolated lymphocytes were all grown in RPMI-1640 supplemented with 10% serum, 1% penicillin‒streptomycin, 25 µl/mL human recombinant IL2 (Stemcell), and 25 µl/mL CD3/CD28 T-cell activator (Stemcell).

### Isolation and identification of sEV

The tissue sEVs were separated as previously described [11]. Briefly, after thawing, the thyroid tissue was very gently dissociated into small pieces and incubated with collagenase D and DNase I for 30 min at 37 °C under mild agitation. Then, a filtration step with a 0.70-µm pore size filter is applied to remove the largest elements. The remaining liquid was differentially centrifuged at 300 × g for 10 min and 2000 × g for 20 min to remove cells and tissue debris at 4 °C. The supernatant was then further centrifuged at 16,500 × g for 20 min and 118,000 × g for 2.5 h at twice to collect sEVs (SW-32Ti) at 4 °C. For T lymphocyte-derived sEVs, media with fetal bovine serum (previously depleted from serum-sEVs) was added, and the cell supernatants were collected after 72 h [19]. The cell supernatant was centrifuged at 300 × g for 3 min and then at 2000 × g for 10 min to discard dead cells, followed by 10,000 × g for 30 min at 4 °C. The supernatants were further ultracentrifuged twice at 100,000 × g and 4 °C for 2 h to collect sEVs [8]. The sEVs were characterized by nanoparticle tracking analysis (NTA), transmission electron microscopy (TEM) and western blot analysis [20]. We have submitted all relevant data of our experiments to the EV-TRACK knowledgebase (ID: EV230028) [21].

### Western blot analysis

Tissue sEV suspensions were harvested in RIPA buffer (Sigma) supplemented with a protease and phosphatase inhibitor cocktail (Thermo) and protein concentration was determined by Pierce™ BCA assay kit (Thermo) according to the manufacturer’s instructions. Proteins (30 μg) from sEV samples or from cellular lysates were migrated on 10% SDS-PAGE gels. Following electrophoresis, proteins were transferred onto PVDF membranes and blocked at room temperature in Tris-buffered saline/0.3% Tween 20 containing 4% BSA. For identification of sEVs, the membranes were then incubated overnight at 4 °C with the specific markers TSG101 (Abcam, ab125011, 1:1000), HSP70 (Abcam, ab181606, 1:1000), CD63 (Santa Cruz Cat, sc-5275, 1:200), CD81 (Huabio, ET1611-87, 1:1000) and calnexin (Proteintech, 10,427–2, 1:500). The ERK1/2 pathway-related proteins RAC1 (Abcam, ab155938, 1:1000), p-ERK1/2 (CST, 4695, 1:1000), Caspases 3 (CST, 9662, 1:1000) and GAPDH (Abcam, ab9485, 1:2500) were also used in western blot (WB) analysis. Horseradish peroxidase-conjugated secondary antibodies, including goat anti-rabbit (Abcam, ab6721, 1:5000) and goat anti-mouse (Abcam, ab6789, 1:5000), were used. The uncropped original pictures of WB are presented in Additional file 1: Fig. S1.

### Total RNA extraction and RNA analyses

Total RNA from cell or tissue sEVs was extracted using TRIzol reagent (Invitrogen) according to the manufacturer’s instructions. The RNA concentration and purity were confirmed using the RNA Nano 6000 Assay Kit of the Agilent Bioanalyzer 2100 System (Agilent Technologies) (Additional file 1: Fig. S2A). The eligible samples of tissue sEV total RNA were used for high-throughput RNA-Seq of miRNAs.

### Library preparation and sequencing

A total amount of 1 ng-500 ng RNA per sample was used as input material for the RNA sample preparations. Sequencing libraries were generated using the QIAseq miRNA Library Kit (Qiagen) following the manufacturer’s recommendations, and index codes were added to attribute sequences to each sample. Reverse transcription primers with unique molecular indices were introduced to analyze the quantification of miRNA expression during cDNA synthesis and polymerase chain reaction (qPCR) amplification. Then, library quality was assessed on the Agilent Bioanalyzer 2100 and quantitative PCR. The clustering of the index-coded samples was performed on an acBot Cluster Generation System using TruSeq PE Cluster Kitv3-cBot-HS (Illumina) according to the manufacturer’s instructions. After cluster generation, the library preparations were sequenced on an Illumina HiSeq platform, and paired-end reads were generated.

### Quantification and differential expression analysis of miRNA

Using bowtie tools soft, the clean reads were aligned with the silva database, GtRNAdb database, Rfam database and Repbase database sequence alignment, filter ribosomal RNA, transfer RNA, small nuclear RNA, small nucleolar RNA, and other non-coding RNA and repeats. The remaining reads were used to detect known miRNAs and new miRNAs predicted by comparison with known miRNAs from miRbase and Human Genome (GRCh38), respectively. The expression matrix of quantified unique molecular indices counts of miRNAs was normalized to counts per million and calculated to relative log expression via the EdgeR package. Differentially expressed miRNAs (DEMs) in tissue sEVs between HT and normal tissue were filtered by** |**log2 (fold change)** |**> 1 and *p* < 0.05. Gene ontology enrichment analysis of the target genes of DEMs was implemented by the topGO R packages. KOBAS software was used to test the statistical enrichment of DEMs in KEGG pathways. Inflammation-related signaling pathways and immune system processes are presented.

### RNA reverse transcription and real-time PCR

The miRCURY LNA™ miRNA PCR Starter Kit was used for quantitative real-time PCR (qRT‒PCR) (Qiagen), and miR-103a-3p was used as an internal control (Additional file 1: Fig. S2B) [22]. Briefly, total sEV RNA (20 ng) was reverse transcribed to cDNA, and qRT‒PCR was conducted in 96-well plates on a CFX96 RT‒PCR detection system. For mRNA analysis, RNA (1 μg) was reverse transcribed to cDNA, and then qRT‒PCR was conducted using a SYBR Green PCR Kit (TaKaRa). GAPDH was used as a reference gene. The expression level was calculated by the 2^−ΔΔCT^ method.

### Confocal microscopy

For sEV view analysis, the isolated tissue sEVs were incubated with fluorescent antibodies of anti-EpCAM (BioLegend) and anti-CD45 (BioLegend) for 1 h at room temperature. Tissue sEVs were imaged by a Nikon A1 confocal microscope (Nikon Instruments). For uptake of sEVs, purified T lymphocyte-derived sEVs (approximately 6.4 × 10^9^ for both normal and HT subjects) were labeled with a PKH67 green fluorescent labeling kit (Sigma‒Aldrich). Then, Nthy-ori-3–1 cells were cocultured with PKH67-labeled sEVs and visualized after 24 h [8, 23].

### Fluorescence in situ hybridization (FISH) and immunohistochemistry (IHC)

FISH was performed on frozen sections by using the 5′- and 3′-digoxigenin-labeled locked nucleic acid probe complementary to the mature miR-142-3p according to the manufacturer’s instructions. The miRNA signal was detected with the Alexa Fluor 488 Tyramide SuperBoost Kit (Invitrogen, Thermo Fisher Scientific) following the manufacturer’s directions. IHC for Treg cells was performed on formalin-fixed, paraffin-embedded tissue sections. Anti-human Foxp3 primary antibody (Biolegend, 320,102, 1:200) and anti-mouse Foxp3 primary antibody (Abcam, ab215206, 1:500) were used. The subsequent steps were performed using the GTVision™ + Detection System/Mo&Rb kit according to the manufacturer’s instructions, which was purchased from Gene Tech Co., Ltd. (Shanghai, China).

### MiRNAs, small interfering RNA, and transfection

The corresponding control mimics, miRNA mimics, control inhibitor, miRNA inhibitor, or small interfering RNA (siRNA) were purchased from Ribo Biotech Co., Ltd. (Guangzhou, China). Cells were seeded into 12-well plates, cultured until 60–70% confluence and then transiently transfected using a Lipofectamine 3000 Transfection Kit (Invitrogen) according to the manufacturer’s instructions. After 48 h of transfection, the cells were harvested and used for further assays. The human thyroid organoid model was constructed as described previously [24]. Briefly, fresh normal thyroid tissue was collected and digested into single cells. The cells were seeded into a 12-well plate in Matrigel with growth factors and transfected at 72 h internal.

### Proliferation and apoptosis

The proliferation of Treg cells was detected by 5,6-carboxyfluorescein diacetate succinimidylester (CFSE) via flow cytometry. For thyrocyte cell proliferation, cells were seeded into regular six-well plates at a density of 2000 cells per well. After 7 days, the resulting colonies were stained with crystal violet. The proliferation of thyroid organoids was measured by the size of organoids after 12 days of culture. The apoptosis of thyrocyte cells was measured by flow cytometry, and the apoptosis of thyroid organoids was measured by fluorescence microscopy using the Annexin V-APC/7-AAD apoptosis kit (MultiSciences).

### T lymphocyte migration and chemokine titration assays

The lower chambers of the Transwell inserts (8 μm pore size, Costar) were filled with 600 μl of cell supernatant from treated Nthy-ori-3–1 cells, while the upper chambers were filled with 100 μl of activated T lymphocytes (5 × 10^6^/mL). Then, the Transwell system was placed in a CO_2_ incubator for 6 h. T lymphocytes that migrated to the lower chamber were counted. Additionally, cell supernatants from treated Nthy-ori-3–1 cells and Treg cells were harvested. CXCL9/10/11 and TGF-β/IL-10 were quantified using enzyme-linked immunosorbent assay (ELISA) kits according to the manufacturer's instructions.

### Dual-luciferase reporter assay

The sequences for *RAC1*-mut and *RAC1*-wt containing the putative binding sites of miR-142-3p were amplified and then cloned into the pmirGLO-control luciferase reporter vectors (Promega). The wt/mut vectors were cotransfected with control mimics and miR-142-3p mimics into 293 T cells in the presence of Lipofectamine 2000 (Invitrogen). At 36 h after cell culture, the cells were lysed, and luciferase activities were assessed.

### In vivo experiments

The 5-week-old littermate non-obese diabetic (NOD).H-2^h4^ female mice obtained from the Jackson Laboratory were randomly divided into two groups using a random-number table and given high-iodine water that contained 0.05% sodium iodide. The two groups were the lentiviral vector (LV) control group (LV-ctrl) and the LV inhibitor of the miR-142-3p group (LV-sponge). The corresponding author was aware of the group allocation at the different stages of the experiment. The animal number is calculated based on our preliminary data at a 2-sided significance of 0.05 and a power of 0.90. Ultimately, we allocated six mice to each group to evaluate the lymphocyte infiltration in thyroiditis and three mice to each group to evaluate the serum TgAb, TPOAb, and Treg cells in the spleen, respectively. The total number of mice used was 18 and there were no exclusions. All mice were treated with lentivirus tail vein intervention (1 × 10^7^ TU each time) as described previously [25]. Mice were sacrificed by iodine feeding for 12 weeks. Thyroid paraffin sections were collected for hematoxylin and eosin (HE) staining. The severity of lymphocyte infiltration in thyroiditis was rated by the collective areas of inflammatory lymphocyte cells as described previously [25]. The levels of serum TgAb and TPOAb were detected by ELISA according to the manufacturer’s instructions. Treg cells in the thyroid were evaluated by IHC. Furthermore, mouse CD4^+^ T lymphocytes were isolated from the spleen, and Treg cells were further detected by staining with anti-mouse Foxp3 PE (Abcam). The level of protein was evaluated by WB analysis. All protocols were in accordance with the guidelines for animal experimentation and were approved by the institutional animal care committee of West China Hospital of Sichuan University (Number: 20220221030). The ARRIVE guidelines were followed (Additional file 2).

### Statistical analysis

Statistical Package for the Social Sciences, version 25.0 (SPSS, Chicago, IL), and GraphPad Prism, version 8.0 (GraphPad Software, Inc.) were used for data analysis. The distribution of data was analyzed by descriptive statistics and the Kolmogorov–Smirnov test, as well as by evaluation of quantile‒quantile plots. Normally distributed quantitative variables are presented as the mean ± standard deviation/standard error and were compared using Student’s *t* test or one-way ANOVA. Nonnormally distributed parameters are expressed as the median, and the two groups were compared using nonparametric Mann‒Whitney *U* tests. Comparisons of categorical variables were performed using the chi-square test or Fisher’s exact test. Pearson correlation coefficients were used to evaluate the correlations between the variables. A receiver operating characteristic (ROC) curve was constructed to determine the predictive efficacy and a logistic regression model was used to determine the most relevant tissue sEV miRNAs for HT. A *P* value < 0.05 indicated statistical significance (^*^P < 0.05, ^**^P < 0.01, ^***^P < 0.001, ^****^P < 0.0001).

## Results

### Increased abundance of tissue sEV miR-142-3p is correlated with clinical features of HT

**Fig. 1:**
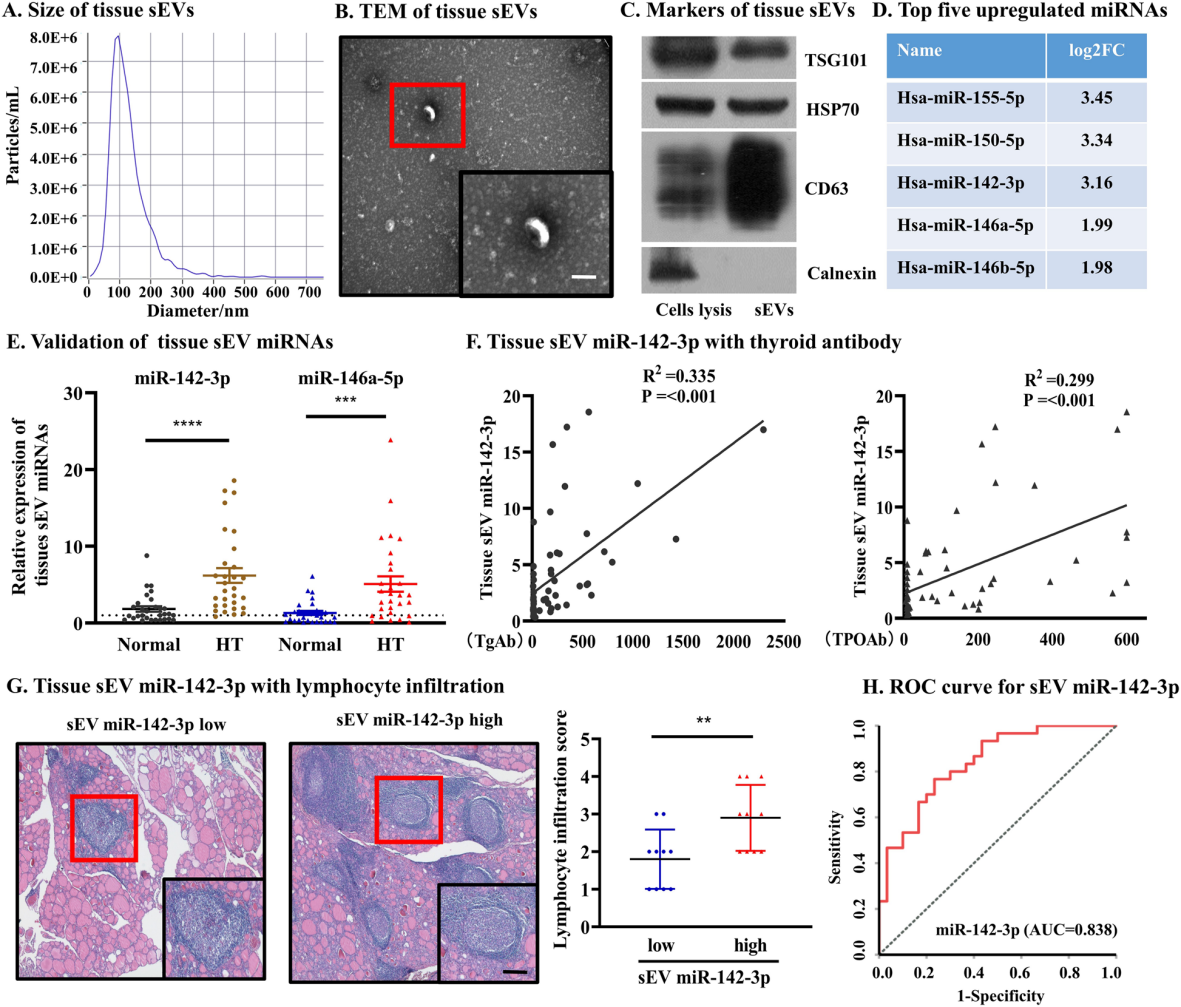
Increased abundance of tissue sEV miR-142-3p is correlated with clinical features of HT. **A**–**C** Tissue sEVs were validated by NTA, TEM (scale bar, 100 nm), and WB.** D** The top five tissue sEV miRNAs expressed in all testing samples. **E** Two tissue sEV miRNAs were further confirmed by qRT‒PCR in a validation set. **F** Tissue sEV miR-142-3p was positively associated with the levels of TgAb and TPOAb.** G** Higher tissue sEV miR-142-3p was accompanied by more severe lymphocyte infiltration in thyroid tissue, scale bar, 100 μm. **H** ROC curve showed that tissue sEV miR-142-3p achieved an AUC of 0.838

To investigate whether tissue sEVs are involved in the pathogenesis of HT, tissue sEVs were first isolated and validated via NTA (Fig. 1A), TEM (Fig. 1B), and the expression of the positive markers TSG101, HSP70, and CD63 without the expression of the negative marker calnexin (Fig. 1C). The deleterious impact of HT tissue sEVs on thyrocyte cell survival was observed in our preliminary analysis (*p* = 0.003, Additional file 1: Fig. S3). Thus, to identify the potential tissue sEV miRNAs associated with HT, we first subjected the 20 tissue samples in the testing set to sEV miRNA sequencing (Additional file 1: Table S1). By DEM analysis, 44 upregulated and 26 downregulated tissue sEV miRNAs were identified in HT (all *p* < 0.05, Additional file 1: Fig. S4A), and enrichment analysis showed that those miRNAs were enriched in many inflammation-related signaling pathways and immune system processes (Additional file 1: Fig. S4B-C). Then, the top five tissue sEV miRNAs expressed in all testing samples were selected (all *p* < 0.05, Fig. 1D and Additional file 3) and further verified in a larger sample set (Additional file 1: Table S1), which showed that two tissue sEV miRNAs (miR-142-3p and miR-146a-5p) were significantly upregulated in HT tissue in comparison with normal tissue (all *p* < 0.001, Fig. 1E and Additional file 1: Fig. S4D).

To identify the most relevant tissue sEV miRNAs to HT, logistic regression was performed (Additional file 1: Table S2), and the results revealed that tissue sEV miR-142-3p is an independent risk factor for HT (*p* = 0.040). Thus, we further assessed the existence of any possible associations between tissue sEV miR-142-3p levels and clinical features. Pearson correlation coefficients showed that tissue sEV miR-142-3p was positively associated with the levels of TgAb (*R*^2^ = 0.335, *p* < 0.001) and TPOAb (*R*^2^ = 0.299, *p* < 0.001) (Fig. 1F and Additional file 1: Fig. S4E). Furthermore, HE staining revealed that higher tissue sEV miR-142-3p levels were accompanied by more severe lymphocyte infiltration in HT tissue (*p* = 0.009, Fig. 1G). Finally, we assessed the discriminating potency between HT and controls of tissue sEV miR-142-3p using ROC curve analysis, which showed that tissue sEV miR-142-3p achieved an AUC of 0.838 (Fig. 1H). Taken together, these results demonstrated that tissue sEV miR-142-3p is highly expressed in HT patients and correlated with higher levels of thyroid antibodies, which led us to hypothesize that tissue sEV miR-142-3p may be involved in the pathogenesis of HT.

### Tissue sEV miR-142-3p was derived from T lymphocytes and can be transferred to thyrocyte cells

**Fig. 2:**
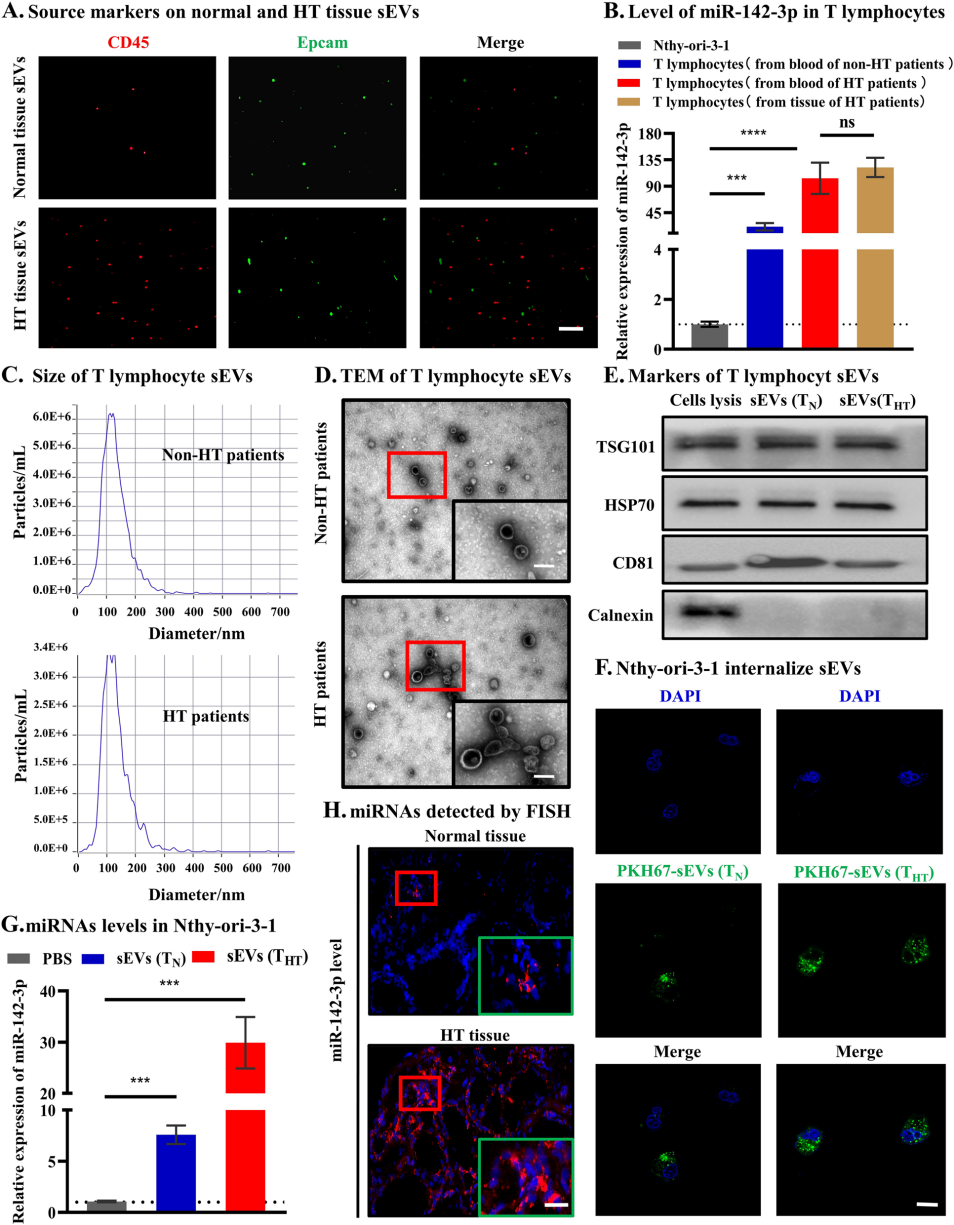
Tissue sEV miR-142-3p was derived from T lymphocytes and can be transferred to thyrocyte cells. **A** HT tissue showed more immune cell-derived sEVs, scale bar, 10 μm. **B** miR-142-3p is highly abundant in T lymphocytes, especially T lymphocytes isolated from HT patients. **C**–**E** T lymphocyte-derived sEVs were validated by NTA, TEM (scale bar, 100 nm) and WB. **F** Confocal microscopy showed that thyrocyte cells can internalize green-labeled T lymphocyte-derived sEVs, scale bar, 20 μm. **G** The expression of miR-142-3p was higher in thyrocyte cells incubated with sEVs than in thyrocyte cells incubated with PBS. **H** FISH showed that miR-142-3p was minimally detected in the thyroid glands of healthy controls but abundantly detected in the thyroid glands of patients with HT, scale bar, 50 μm

To explore parental cells of tissue sEV miR-142-3p, we first analyzed the composition of tissue sEVs in HT tissue. HT tissue mainly consists of infiltrated immune cells and thyrocyte cells [26], which are considered the parental cells of tissue sEVs. Thus, the relative levels of sEV with the indicated surface markers (CD45 for immune cells and EpCAM for thyrocyte cells [27]) were further measured. Compared with normal tissue, HT tissue showed more immune cell-derived sEVs (Fig. 2A). Additionally, miR-142-3p is reported to be primarily expressed in the hematopoietic compartment, particularly in T lymphocytes (Additional file 1: Fig. S5A) [28–30]. We therefore hypothesized that the increase in tissue sEV miR-142-3p in HT may result from T lymphocytes. To explore this hypothesis, we first isolated human CD4^+^ T lymphocytes (the most common immune cells in HT [1, 26]) and found that miR-142-3p is indeed much more abundant in T lymphocytes than in thyrocyte cells, especially T lymphocytes isolated from HT patients (*p* < 0.001, Fig. 2B). Furthermore, we isolated T lymphocyte-derived sEVs (Fig. 2C–E) and examined the miRNA content. MiR-142-3p was found to be present in T lymphocyte-derived sEVs, especially those released by T lymphocytes from HT patients (*p* < 0.001, Additional file 1: Fig. S5B). We next investigated whether these native T lymphocyte-derived sEVs can be efficiently internalized by thyrocyte cells. After an overnight incubation, PKH67-labeled sEVs were found to be taken up by the thyrocyte cell line Nthy-ori-3–1 (Fig. 2F). To further investigate whether this T lymphocyte-derived sEV miR-142-3p can be transferred to Nthy-ori-3–1, T lymphocytes were first transfected with the miR-142-3p mimics. sEVs were then isolated from mimic-transfected T lymphocytes (data not shown) and cocultured with thyrocyte cells, which showed that T lymphocyte-derived sEVs can deliver miR-142-3p to upregulate the expression of miR-142-3p in thyrocyte cells (all *p* < 0.001, Fig. 2G). Then, we validated the expression of miR-142-3p in clinical samples (thyroid glands of healthy controls and patients with HT). FISH showed that miR-142-3p was minimally detected in the thyroid glands of healthy controls but abundantly detected in the thyroid glands of patients with HT (Fig. 2H). The above results indicated that the expression of miR-142-3p was higher in T lymphocytes in HT patients than in healthy controls and that miR-142-3p in these T lymphocytes can be transferred to thyrocyte cells via tissue sEVs.

### MiR-142-3p is highly expressed in T lymphocytes and can induce Treg function defect

**Fig. 3:**
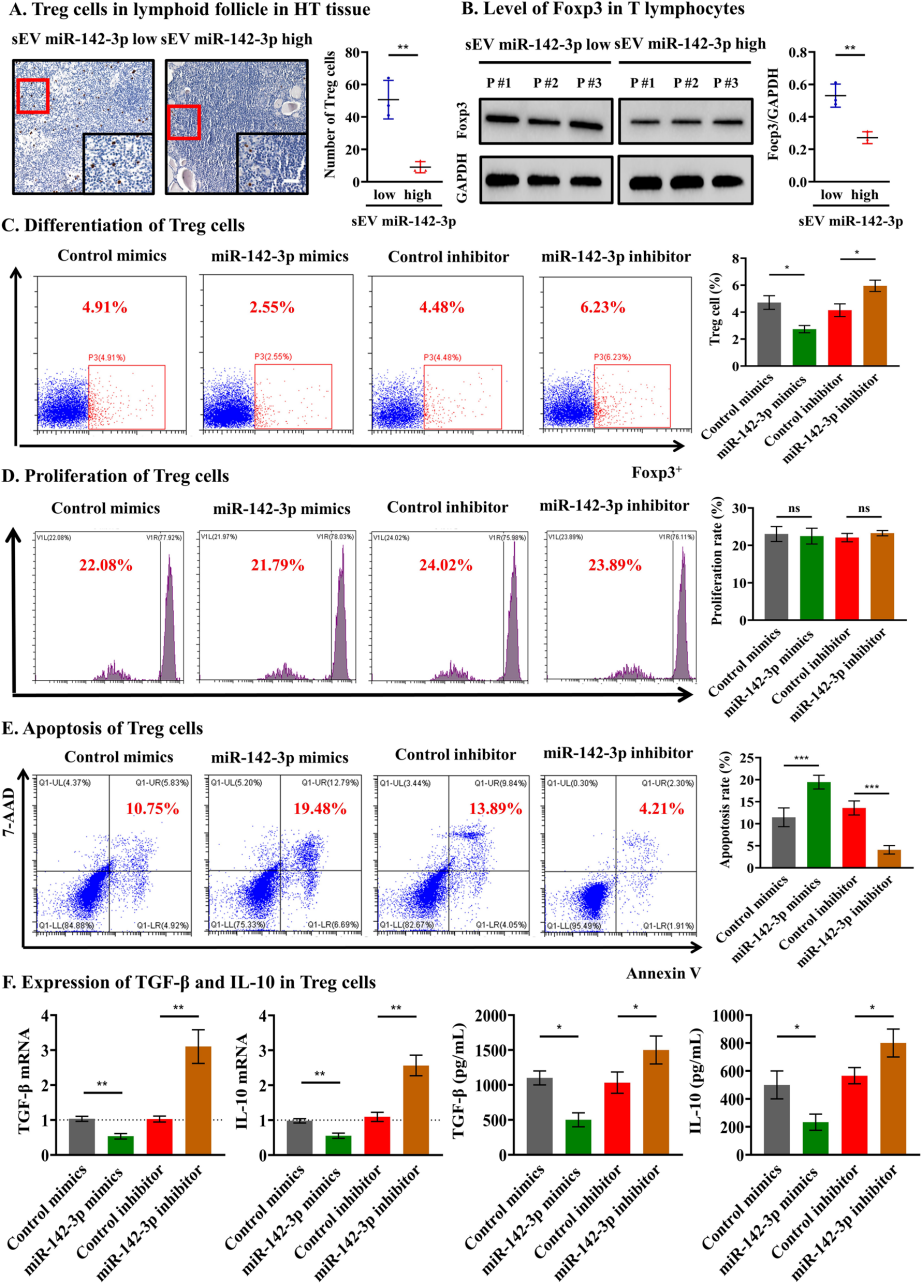
MiR-142-3p highly expressed in T lymphocytes can induce Treg function defects. **A** Higher tissue sEV miR-142-3p levels were accompanied by fewer Treg cells in HT tissue, scale bar, 100 μm. **B** Higher miR-142-3p in T lymphocytes indicated less Foxp3 expression. **C** The induced expression of miR-142-3p in T lymphocytes inhibits Treg differentiation. **D** miR-142-3p has no effect on the proliferation of Treg cells. **E** Apoptosis of Treg cells was significantly increased after treatment with mimics and decreased after treatment with inhibitor. **F** Cytokine experiments showed that overexpression of miR-142-3p resulted in significant decreases in secretion of the anti-inflammatory factors TGF-β and IL-10

Previous studies have reported that miR-142-3p is correlated with Treg function [31–33]. Our preliminary analysis also showed that higher tissue sEV miR-142-3p levels were accompanied by fewer Treg cells in HT tissue (*p* = 0.004, Fig. 3A), and higher miR-142-3p in T lymphocytes indicated less Foxp3 expression (*p* = 0.005, Fig. 3B), which advises us to examine whether the highly expressed miR-142-3p in T lymphocytes can control Treg biological properties in HT. Thus, the level of miR-142-3p in T lymphocytes was directly interfered with by miR-142-3p mimics and inhibitor ([*p* = 0.003 and 0.006, respectively], Additional file 1: Fig. S5C). To explore the effect of miR-142-3p on Treg differentiation, flow cytometry analysis was performed, and the results showed that the induced expression of miR-142-3p in human CD4^+^ T lymphocytes downregulated the proportion of Treg cells compared with that observed for the control mimics treatment, whereas the inhibition of miR-142-3p increased the number of Treg cells ([ *p* = 0.016 and 0.020, respectively], Fig. 3C). Subsequently, Treg cells were purified from human CD4^+^ T lymphocytes. We further observed that the proliferation of Treg cells was not changed as the miR-142-3p mimics or inhibitor was added (all *p* > 0.05, Fig. 3D), but the apoptosis of Treg cells was significantly increased after treatment with mimics and decreased after treatment with inhibitor ([*p* = 0.006 and < 0.001, respectively], Fig. 3E). Furthermore, a series of cytokine experiments found that overexpression of miR-142-3p resulted in significant decreases in TGF-β and IL-10 secretion, which can exert immunosuppressive effects (all *p* < 0.05, Fig. 3F). These results suggested that miR-142-3p highly expressed in T lymphocytes can induce Treg function defect in HT.

### T lymphocyte-derived tissue sEV miR-142-3p promotes thyrocyte destruction

**Fig. 4:**
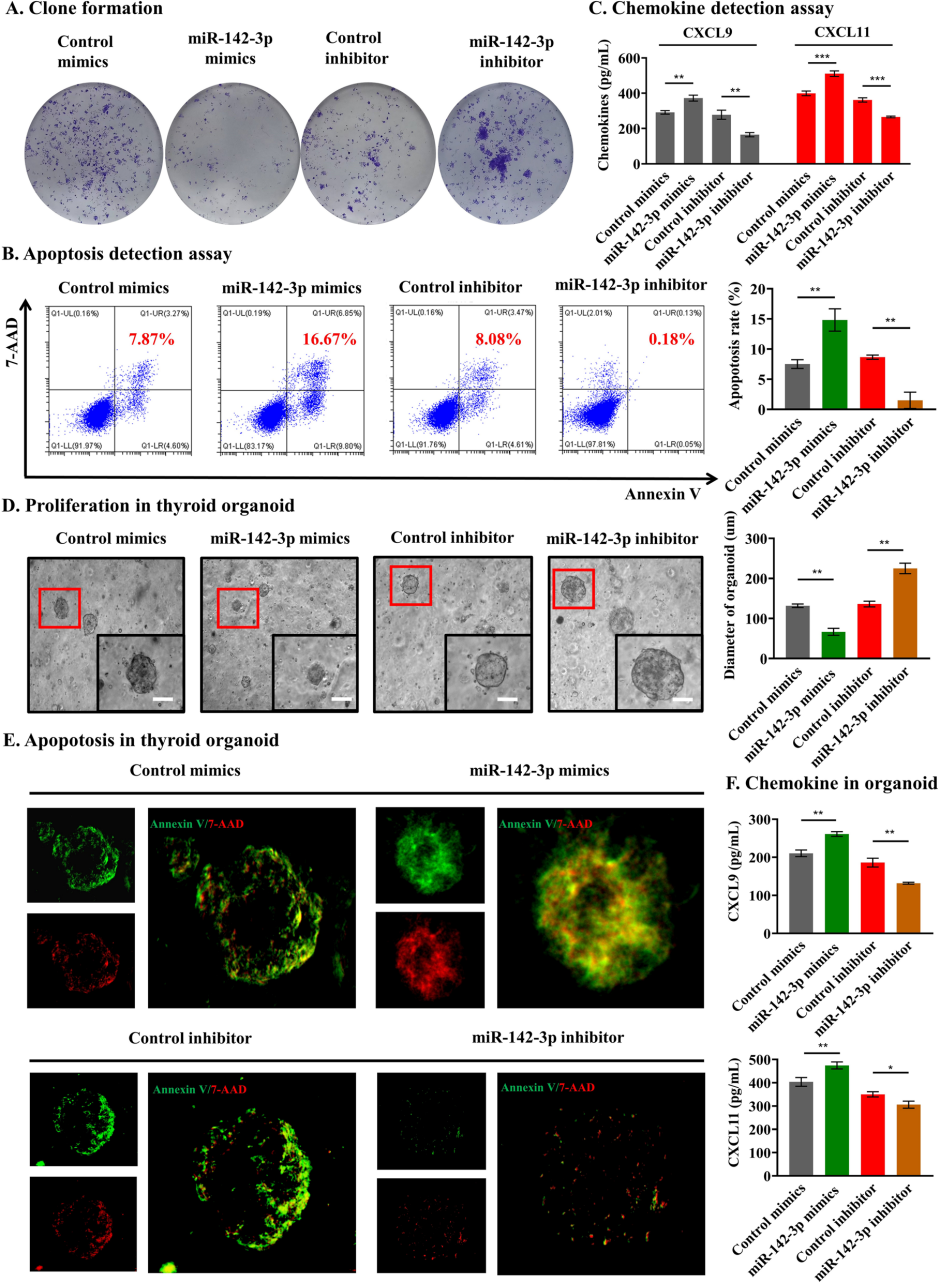
T lymphocyte-derived tissue sEV miR-142-3p promotes thyrocyte destruction. **A** Colony formation assays showed that miR-142-3p suppressed thyrocyte cell proliferation. **B** Flow cytometry suggested that mimic-treated thyrocytes underwent a significant increase in apoptosis, whereas the inhibition of miR-142-3p decreased the apoptosis of thyrocytes.** C** Mimic-treated thyrocyte cells can secrete more CXCL9/11, whereas the inhibition of miR-142-3p decreased the secretion of CXCL9/11. **D** The size of the organoid decreased after transfection with the miR-142-3p mimics and increased after transfection with the inhibitor; scale bar, 100 μm. **E** Apoptosis was increased in organoids after transfection with the mimics and decreased after transfection with the inhibitor.** F** The chemokine assay showed that mimic-treated thyroid organoids secreted more CXCL9/11. In contrast, the miR-142-3p inhibitor decreased the secretion of CXCL9/11

To determine whether tissue sEV-mediated miR-142-3p delivery contributes to the deleterious effect on thyrocyte activities, the miR-142-3p level in thyrocyte cells was also directly interfered with by mimics and inhibitor ([*p* = 0.002 and 0.006, respectively], Additional file 1: Fig. S6A). The clone formation assay showed that miR-142-3p overexpression significantly suppressed the proliferation of thyrocyte cells (Fig. 4A). In contrast, treatment with the miR-142-3p inhibitor significantly promoted proliferation. Flow cytometry suggested that mimic-treated thyrocyte cells underwent a significant increase in apoptosis, whereas the inhibition of miR-142-3p decreased the apoptosis of thyrocyte cells ([*p* = 0.021 and 0.007, respectively], Fig. 4B). Furthermore, we found that the cell supernatant from mimic-treated Nthy-ori-3–1 cells presented a higher attraction ability for T lymphocytes (all *p* < 0.05, Additional file 1: Fig. S6B). CXCL9/10/11 has been reported to be increased in HT and to play an important role in attracting T lymphocytes [7]. Therefore, we detected chemokines and found that mimic-treated thyrocytes secreted more CXCL9/11 (all *p* < 0.05, Fig. 4C and Additional file 1: Fig. S6C). To better simulate the conditions in vivo, three-dimensional tests were also conducted. In agreement with our above findings, thyroid organoid growth assays revealed that the size of the organoids decreased after transfection with the miR-142-3p mimics and increased after transfection with the inhibitor ([*p* = 0.003 and 0.004, respectively], Fig. 4D). Additionally, the apoptosis of thyroid organoids was increased after transfection with miR-142-3p mimics and decreased after transfection with the inhibitor (Fig. 4E). The chemokine assay showed that mimic-treated thyroid organoids also secreted more CXCL9/11 (all *p* < 0.05, Fig. 4F). To further confirm that the transfer of miR-142-3p from T lymphocytes to thyrocyte cells contributes to the destruction induced by tissue sEVs, we used an inhibitor to block the action of miR-142-3p and found that the miR-142-3p inhibitor prevented apoptosis induced by HT tissue sEVs (Additional file 1: Fig. S3). Taken together, these results demonstrate that T lymphocyte-derived tissue sEV-mediated miR-142-3p transfer is involved in promoting thyrocyte destruction.

### MiR-142-3p regulated the ERK1/2 pathway by targeting RAC1 in thyrocyte cells

**Fig. 5:**
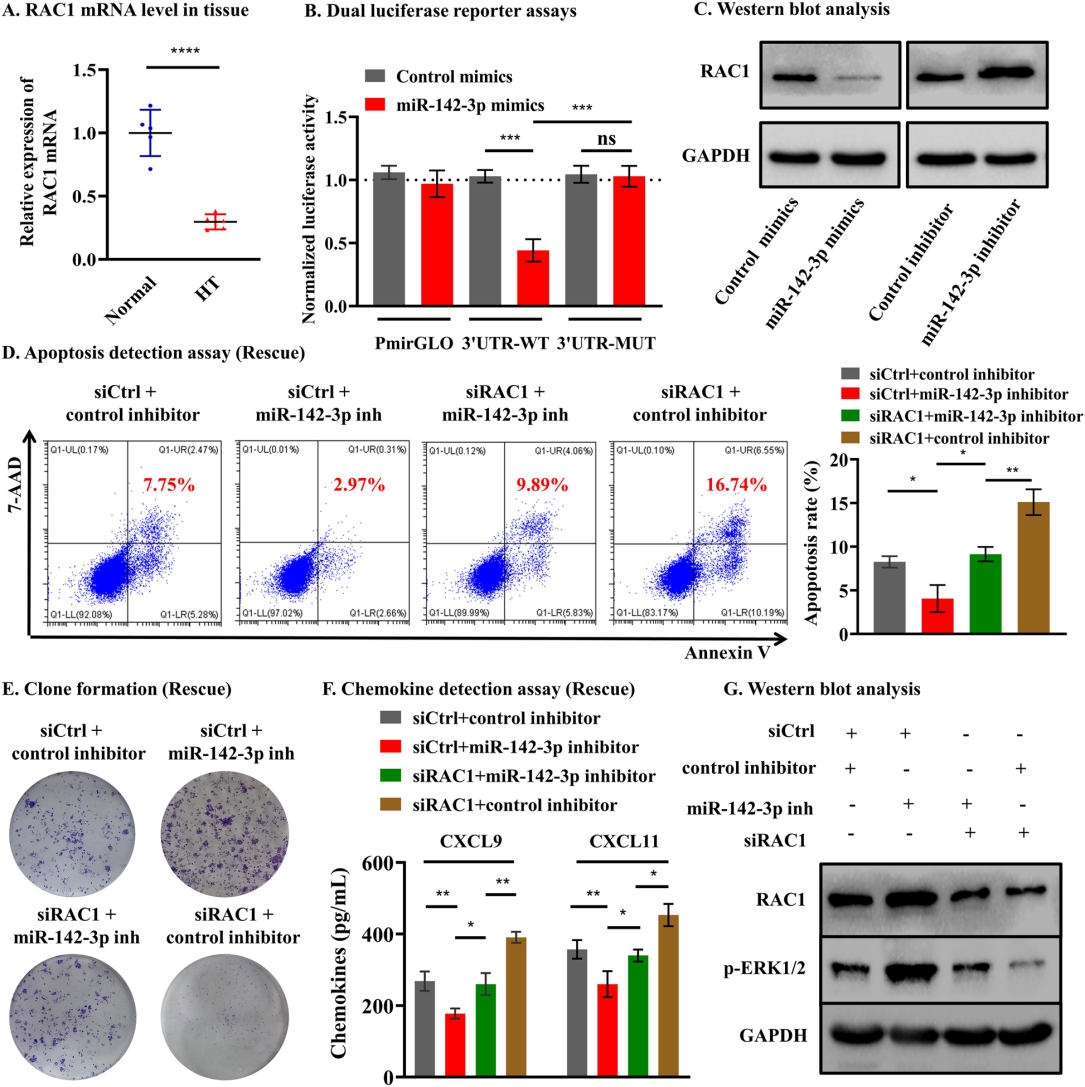
MiR-142-3p regulated the ERK1/2 pathway by targeting *RAC1* in thyrocyte cells. **A** qRT‒PCR showed that the level of *RAC1* mRNA was significantly decreased in HT tissue compared with normal tissue. **B** Dual luciferase reporter assays showed that miR-142-3p could significantly reduce wild-type *RAC1* luciferase activity. **C** WB analysis showed that miR-142-3p could inhibit *RAC1* expression at the protein level. **D**–**F** Downregulation of *RAC1* expression effectively reverses miR-142-3p inhibitor-induced apoptosis, proliferation, and secretion of CXCL9/11 in thyrocyte cells.** G** WB analysis showed that miR-142-3p regulates ERK1/2 pathway-related proteins via *RAC1*

Given that thyrocyte destruction is the main pathophysiological process in HT, we next explored the mechanism by which miR-142-3p modulates the destruction of thyrocyte cells. The potential mRNA targets of this miRNA were predicted by four public bioinformatics algorithms, and 14 potential targets were identified (Additional file 1: Fig. S7A). Among them, *RAC1* was found to be involved in autoimmune diseases by regulating ERK1/2 signaling [23, 34]. Thus, we hypothesized that T lymphocyte-derived sEV miR-142-3p may also affect thyrocyte cell function by targeting *RAC1* to inhibit the ERK1/2 signaling pathway. qRT‒PCR indeed showed that the level of RAC1 mRNA was significantly decreased in HT tissue in comparison with normal tissue (*p* < 0.001, Fig. 5A). To further determine whether *RAC1* is a direct target of miR-142-3p, a predicted binding site was confirmed in the 3′-UTR of *RAC1* mRNA (Additional file 1: Fig. S7B). Then, the luciferase reporter assay showed that miR-142-3p could significantly reduce wild-type *RAC1* luciferase activity (*p* < 0.001, Fig. 5B). WB analysis confirmed that the expression of *RAC1* dramatically changed in the miR-142-3p mimics and inhibitor groups compared with that in the control mimics and control inhibitor groups, respectively (Fig. 5C). Thus, these results suggested that miR-142-3p directly targets *RAC1*.

To determine the functional role of *RAC1* in the miR-142-3p-induced effect on thyrocyte cells, the best siRNA for subsequent experiments was selected (all *p* < 0.001, Additional file 1: Fig. S7C). A rescue experiment was conducted, and the results revealed that thyrocyte cells cotransfected with inhibitor and si-*RAC1* exhibited a higher apoptosis rate (all *p* < 0.05, Fig. 5D), fewer clones (Fig. 5E) and CXCL9/11 secretion (all *p* < 0.05, Fig. 5F) than cells transfected with inhibitor alone. The data above showed that knockdown of *RAC1* could effectively reverse miR-142-3p inhibitor-induced functional changes. Then, we detected ERK1/2 pathway-related proteins and found that the effects of the miR-142-3p inhibitor on p-ERK1/2 were attenuated by silencing *RAC1* (Fig. 5G). Taken together, our results suggested that miR-142-3p can regulate the ERK1/2 signaling pathway by targeting *RAC1* in thyrocyte cells.

### Injection of miR-142-3p sponge prevents HT development in NOD.H-2^h4^ mice

**Fig. 6:**
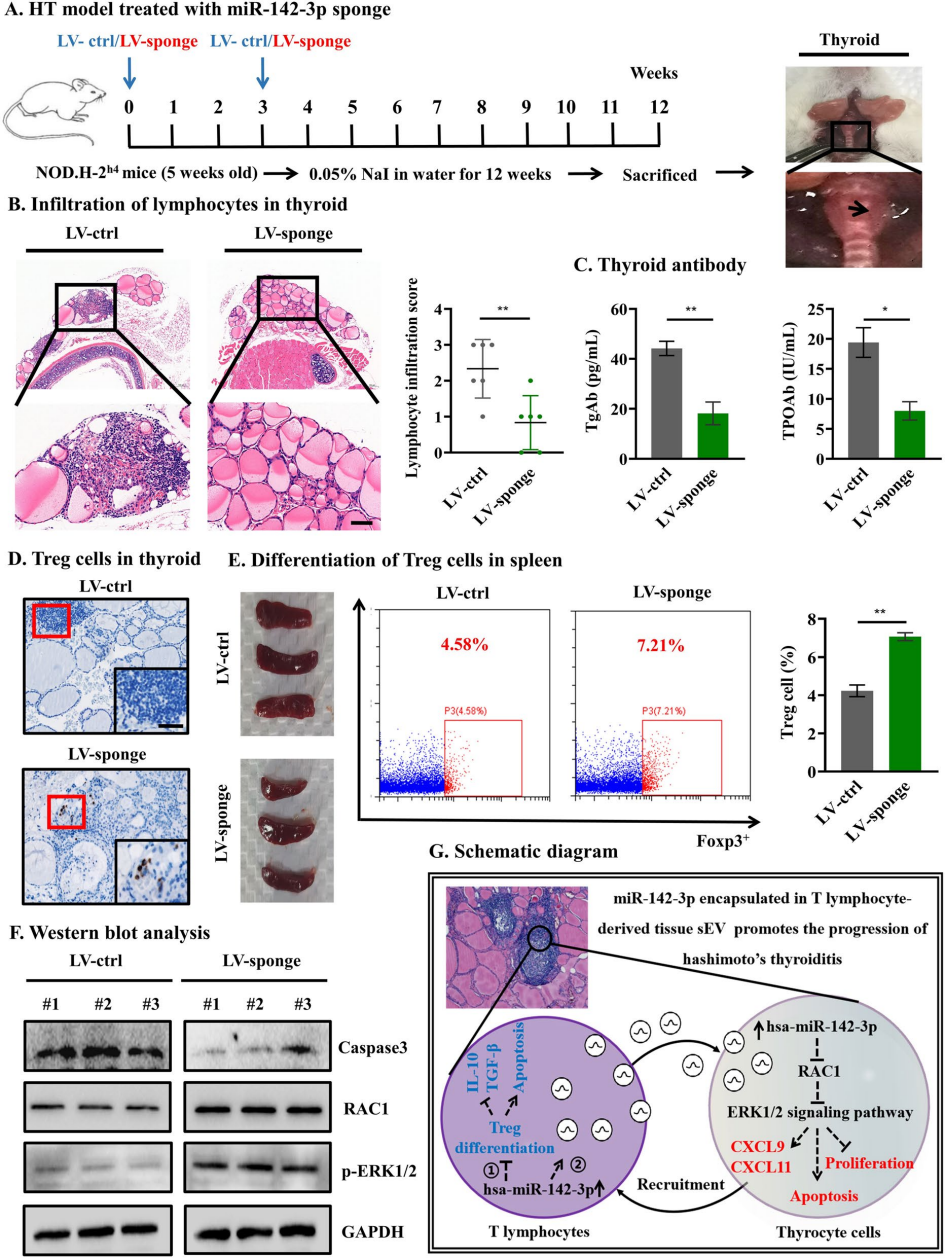
Injection of miR-142-3p sponge prevents HT development in NOD. H-2.^h4^ mice. **A** The mode diagram of LV administration. **B** The scoring of thyroid inflammation in the LV-sponge group was significantly decreased, scale bar, 100 μm. **C** The serum TgAb and TPOAb titers of the LV-sponge group were significantly lower than those of the LV-ctrl group. **D** IHC showed that the Treg cells in the LV-sponge group were higher than those in the LV-ctrl group in the thyroid, scale bar, 50 μm. **E** The mice treated with LV-sponge exhibited a higher Treg percentage than control mice. **F** Caspase3 expression level in thyroid was decreased, but the protein levels of RAC1 and p-ERK1/2 were increased after treatment with LV-sponge compared with LV-ctrl; **G** Schematic diagram (①: MiR-142-3p highly expressed in T lymphocytes induces Treg function defect; ②: T lymphocyte-derived tissue sEV miR-142-3p promotes thyrocyte destruction through the *RAC1*-mediated ERK1/2 signal)

To assess the pathophysiological relevance of tissue sEV-mediated miR-142-3p signaling in vivo, LV-ctrl and LV-sponge were synthesized and injected into NOD.H-2^h4^ mice (Fig. 6A). Thyroid inflammation was determined according to the lymphocyte infiltration area using HE staining of the thyroid gland. Compared with that in the LV-ctrl group, the thyroid inflammation score in the LV-sponge group was significantly decreased (0.83 ± 0.82 vs 2.33 ± 0.75, *p* = 0.008) (Fig. 6B). Consistent with the inflammatory score of the thyroid, the serum TgAb and TPOAb titers of the LV-sponge group were significantly lower than those of the LV-ctrl group (18.15 ± 7.88 vs 44.18 ± 4.95, *p* = 0.008; 8.00 ± 2.65 vs 19.40 ± 4.28, *p* = 0.017) (Fig. 6C). Furthermore, IHC showed that the Treg cells in the LV-sponge group were higher than those in the LV-ctrl group in the thyroid (Fig. 6D). Additionally, we used flow cytometry to assess the frequencies of Treg cells in mouse CD4^+^ T lymphocytes isolated from the spleen. We found that mice treated with LV-sponge exhibited a higher Treg percentage than control mice (7.07 ± 0.21 vs 4.23 ± 0.31, *p* = 0.002) (Fig. 6E). Finally, the expression level of proteins in the thyroid was detected by WB analysis. The results showed that the Caspase3 expression level in the thyroid was decreased, and the protein levels of RAC1 and p-ERK1/2 were increased after treatment with LV-sponge compared with LV-ctrl (Fig. 6F). These in vivo data support that the inactivation of miR-142-3p can protect NOD.H-2^h4^ mice against HT development by alleviating Treg function defect and thyrocyte destruction. Taken together, both the human and mouse model results indicated the important role of miR-142-3p in the pathogenesis of HT (Fig. 6G), which may provide relevant implications for the design of approaches to prevent HT development.

## Discussion

The hallmark of HT is the autoimmune attack of thyrocyte cells, but the events that ultimately result in the hypothyroidism remain to be fully established. In our current study, we provide a novel immune-related mechanism that tissue sEV-mediated transfer of miRNAs from T lymphocytes to thyrocyte cells contributes to the development of HT. sEVs have been reported to participate in the pathogenesis of several autoimmune diseases [8, 9, 23] which can stimulate specific signal transduction pathways, involve in the formation of immune complexes and synapses, and contribute to the maintenance of the inflammatory status. The exposure of thyrocyte cells to HT tissue sEVs can induce their apoptosis, an essential characteristic of the autoimmune attack, which is considered to be the consequence of inappropriate expression of cell death-related molecules induced by tissue sEVs.

Tissue sEVs have garnered increasing attention in the recent years due to several favorable advantages, such as a more accurate representation of pathophysiologic state while retaining the cell properties, minimal contaminants with a single-tissue source, and allowing the analysis of the temporal-spatial heterogeneity of the tissue microenvironment [10, 11]. The components of tissue sEVs include lipids, proteins, and nucleic acids, in particular miRNAs [10]. Our data provide strong evidence indicating that the up-regulated miR-142-3p in T lymphocytes can be shuttled to thyrocyte cells by tissue sEV and overexpression of this miRNA is sufficient to induce Treg function defect. Numerous studies in autoimmune diseases, including HT [1, 6], have reported a decrease and impaired function of Treg cells which are known for their important role in attenuating the immune response. Yang et al. [35] reported that reduction in the suppressive capacity of Treg cells in HT patients is regulated by SIRT1-mediated abnormal Foxp3 acetylation. The higher expression of miR-142-3p in T lymphocytes may explain, at least in part, the alteration in the number and function of Treg cells in HT from a new perspective. Under inflammatory conditions, thyrocyte cells are known to undergo apoptosis and produce chemokines, such as CXCL9/10/11 [7]. In agreement with reports in other cell types, the similar pro-apoptotic properties of sEV miR-142-3p from T lymphocytes were observed on pancreatic β cells [8] and endothelial cells [23] in type 1 diabetes and vasculitis, respectively, which suggests that sEV miR-142-3p may have a common mode of action in inflammatory status. Besides, the induction of CXCL9/11, two potent leukocyte chemoattractant molecules, suggests that the release of sEV miR-142-3p by T lymphocytes may result in the triggering of an inflammatory cascade that will induce the recruitment of T lymphocytes at the inflammatory site, thus resulting in the amplification and the maintenance of inflammation conditions.

We were able to verify this novel sEV-mediated cell-to-cell communication mode for HT development in vivo. However, it remains to be determined whether the protective effect of the miRNA sponge can prevent the initial stages of HT. Since an efficient delivery of miRNAs involves the close proximity of the lymphocytes and thyrocyte cells, it is unlikely that this event constitutes the initial trigger of the disorder. Once viral antigens, autoantigens, or other inflammatory mediators initiate the autoimmune response and the thyroid develops immune cell infiltration, the production of chemokine by the thyroid cell via miRNA transfer may contribute to maintain and further exacerbate the inflammatory response. This scenario is consistent with the reduced immune infiltration of the thyroid observed in mice treated with the miRNA sponge. However, the accurate impact of sEV-mediated miRNA transfer on the different stages of the disorders remains to be defined, which may be elucidated by large cross-sectional studies monitoring the impact of the miRNA sponge on HT progression during the natural course.

The specific mechanisms through which the sEVs of T lymphocytes induce thyrocyte cell damage were further unraveled in this study. Our investigation of *RAC1*, a validated miR-142-3p target, uncovered an essential role for the miR-142-3p-*RAC1* axis in the regulation of HT microenvironment. *RAC1* is reported to be a key regulator of NADPH-dependent membrane oxidase, a prominent source of reactive oxygen species, thus having a central role in the inflammatory response [36]. The implications suggested by our findings for *RAC1* as a negative regulator of thyrocyte cell destruction is consistent with the conclusions of a previous report [37]. In addition, the down-regulation of *RAC1* was linked with ERK1/2 pathways which favor the protection of thyrocyte cells [38] and other cell types [23] from inflammation-induced apoptosis. A recent study demonstrated that a Chinese patent medicine decreased both levels of TgAb and TPOAb, and alleviated lymphocytic infiltration by regulating expressions of p-ERK1/2 in a rat model [39], which further highlighted the crucial role of ERK1/2 signals in the HT progression.

The sEV-mediated dialogue between lymphocytes and thyrocyte cells may operate in both directions. Thyrocyte-derived sEVs were reported to stimulate strong T lymphocyte responses via the antigen-presenting cells [40]. In our study, we focused on miRNAs transferred from T lymphocytes to thyrocyte cells. However, the similar miRNA content may exist in sEVs released from other immune cells invading the thyroid. We believe that the delivery of miRNAs to thyrocyte cells during HT progression is mainly promoted by the close proximity of the activated T lymphocytes that infiltrate the thyroid. Indeed, a higher abundance of miR-142-3p was observed in active T lymphocyte-derived sEVs [41]. It is possible that the presence of autoreactive T lymphocyte receptors or other specific components at the sEV surface of infiltrating immune cells may facilitate the fusion with the thyrocyte cells and the delivery of the miRNAs. Moreover, T lymphocyte-derived sEVs may contain additional cargos potentially influencing the activity of the target cells, including other RNAs, proteins, and lipids. A better understanding of the cargo they deliver may shed new light on the pathogenesis of autoimmune disorders.

Inevitably, our study also had some limitations. The main limitation of this study is the fact that the efficacy of the treatment at different stages of the HT was not investigated. Although injection of miR-142-3p sponge prevents HT development, whether the treatment would be equally efficient after intra-thyroid infiltration is unknown. This information is essential for evaluating the translational potential for human HT of strategies targeting the sEV-mediated process. Besides, miR-142-3p may target multiple genes which are involved in regulating thyrocyte activities. Here, we investigated only the effect of RAC1 not including other genes.

## Conclusions

In summary, our results established that tissue miR-142-3p, shuttled from T lymphocytes to thyrocyte cells, is an essential regulator of Treg function and thyrocyte activities in the pathogenesis of HT. Future strategies to avert the development of HT may need to consider the existence of this newly discovered cell-to-cell signaling mechanism.

## Supplementary Information

**Additional file 1: Table S1.** The baseline demographics and clinical characteristics of enrolled subjects. **Table S2**. Univariate and multiple analysis for difference miRNAs. **Fig S1.** The uncropped original western blots from the main figures. **Fig S2.** The lowest RNA concentration of tissue sEV total RNA and levels of tissue sEV miR-103a-3p. **Fig S3.** Tissue sEVs isolated from HT patients promote apoptosis of thyrocyte cells, which can be prevented by a miR-142-3p inhibitor. **Fig S4.** Biological function enrichment analysis and validation of tissue sEV miRNAs. **Fig S5.** The levels of miR-142-3p in specific immune cell types and T lymphocyte-derived sEVs. **Fig S6.** The miR 142-3p levels after transfection with mimics or inhibitor in thyrocyte cells, T lymphocyte migration and the effect of miR-142-3p on CXCL10 secretion. **Fig S7.** Predicted target and inhibition efficiency of siRNA for RAC1.

**Additional file 2.** The ARRIVE guidelines.

**Additional file 3.** The DEM expressed in all testing samples.

## Data Availability

The raw data regarding RNA sequencing can be found in online repositories: https://www.ebi.ac.uk/biostudies/arrayexpress/studies/E-MTAB-12659.

## Abbreviations

DEMs: Differentially expressed miRNAs
FISH: Fluorescence in situ hybridization
FT3: Free thyroxine 3
FT4: Free thyroxine 4
HE: Hematoxylin and eosin
HT: Hashimoto’s thyroiditis
IHC: Immunohistochemistry
LV: Lentiviral vector
miRNAs: MicroRNAs
NOD: Non-obese diabetic
NTA: Nanoparticle tracking analysis
ROC: Receiver operating characteristic
sEVs: Small extracellular vesicles
siRNA: Small interfering RNA
TEM: Transmission electron microscopy
TgAb: Thyroglobulin antibody
TPOAb: Thyroperoxidase antibody
TSH: Thyroid-stimulating hormone
WB: Western blot
