# Supplementary material for: miR-142-3p encapsulated in T lymphocyte-derived tissue small extracellular vesicles induces Treg function defect and thyrocyte destruction in Hashimoto’s thyroiditis
Source: BMC Med. 2023 Jun 6;21:206. doi: 10.1186/s12916-023-02914-7 (PMC10242775; doi:10.1186/s12916-023-02914-7)

Table S1. The baseline demographics and clinical characteristics of enrolled subjects.

Variables	Testing set			Validation set		
	HT（n=10）	Normal（n=10）	P value	HT（n=30）	Normal（n=30）	P value
Age (years)	47.5 ± 11.6	47.1 ± 11.9	0.532	47.1 ± 11.2	46.4 ± 12.1	0.127
Sex (male/female)	4/6	4/6	-	9/21	9/21	-
BMI（kg/m^2）	23.1 ± 2.8	22.9 ± 2.1	0.216	23.4 ± 3.4	23.1 ± 2.7	0.178
TSH（mIU/L）	2.8（0.7-3.8）	2.4（1.2-3.6）	0.187	2.2（0.1-6.6）	2.0（0.2-5.4）	0.467
FT3（pmol/L）	4.2（3.7-4.5）	4.3（3.5-4.3）	0.623	4.7（3.4-7.5）	4.5（3.2-5.3）	0.381
FT4（pmol/L）	15.1（14.2-17.1）	15.4（13.9-18.4）	0.327	15.2（12.8-33.0）	15.2（10.9-23.4）	0.169
TgAb（IU/mL）	209.5（58.1-4000）	38.9（11.2-112）	***<0.001***	253（115-2298）	13.5（10.1-73.4）	***<0.001***
TPOAb（IU/mL）	187.5（60.2-600）	17.6（9.0-32）	***<0.001***	204（44-600）	9（9-16.2）	***<0.001***

HT: Hashimoto thyroiditis; BMI: body mass index; TSH: thyroid stimulating hormone; FT3: free T3; FT4: free T4; TgAb: thyroglobulin antibody; TPOAb:thyroperoxidase antibody.

Table S2. Univariate and multiple analysis for difference miRNAs.

miRNAs	Univariate		Multivariate	
	OR (95%CI)	P value	OR (95%CI)	P value
miR-142-3p	1.613 (1.185-2.195)	***0.002****	1.437 (1.016-2.032)	***0.040****
miR-146a-5p	1.649 (1.176-2.311)	***0.004****	1.372 (0.965-1.951)	0.078

CI: confidence interval; OR: Odds ratio; *****statistically significant difference.

Figure S1. The uncropped original western blots from main figures.

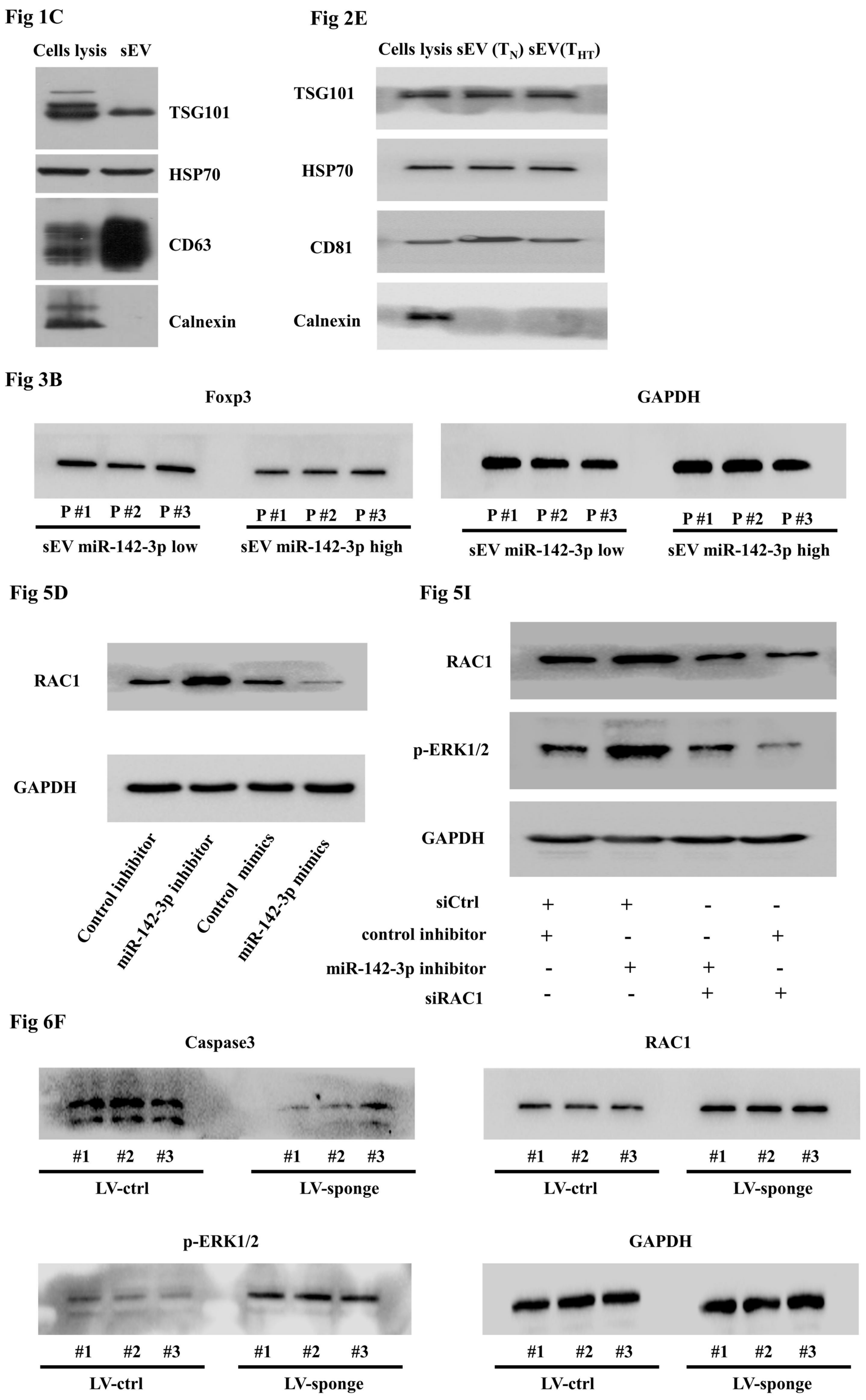

Figure S2. The lowest RNA concentration of tissue sEV total RNA and levels of tissue sEV miR-103a-3p

A. Lowest RNA concentration in sEVs isolated from HT tissue and normal tissue

B. Levels of tissue sEV miR-103a-3p

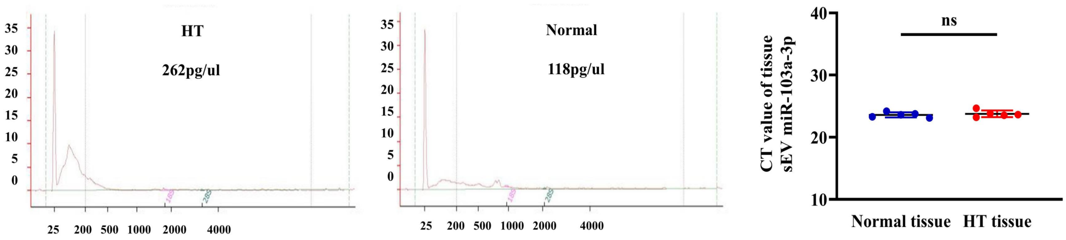

Figure S3. Tissue sEVs isolated from HT patients promotes apoptosis of thyrocyte cells which can be prevented by miR-142-3p inhibitor

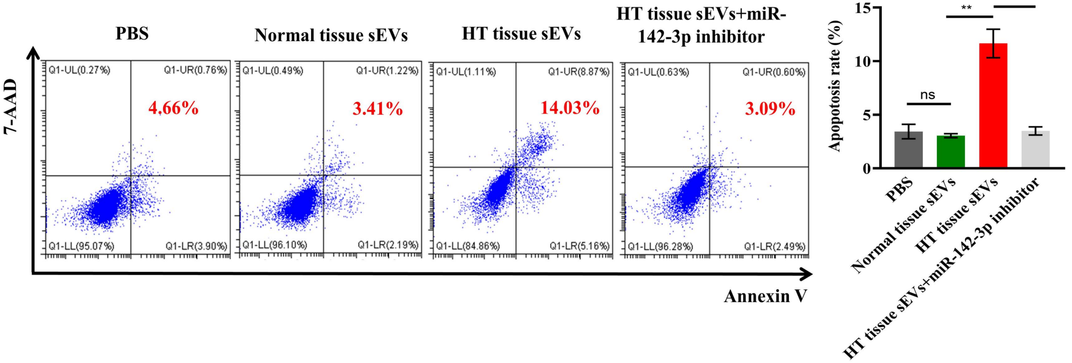

Figure S4. Biological function enrichment analysis and validation of tissue sEV miRNAs

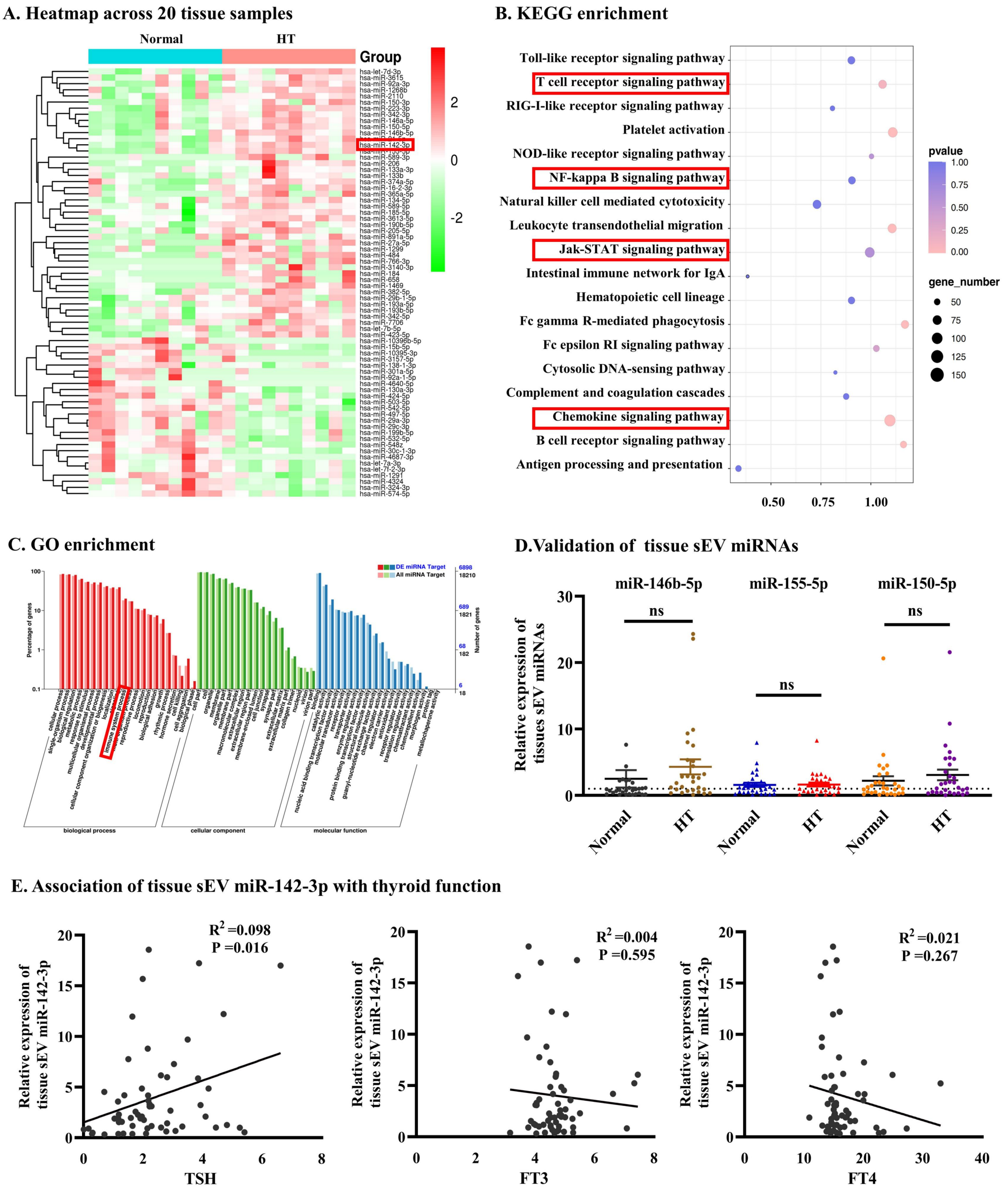

Figure S5. The levels of miR-142-3p in specific immune cell types and T lymphocyte-derived sEV

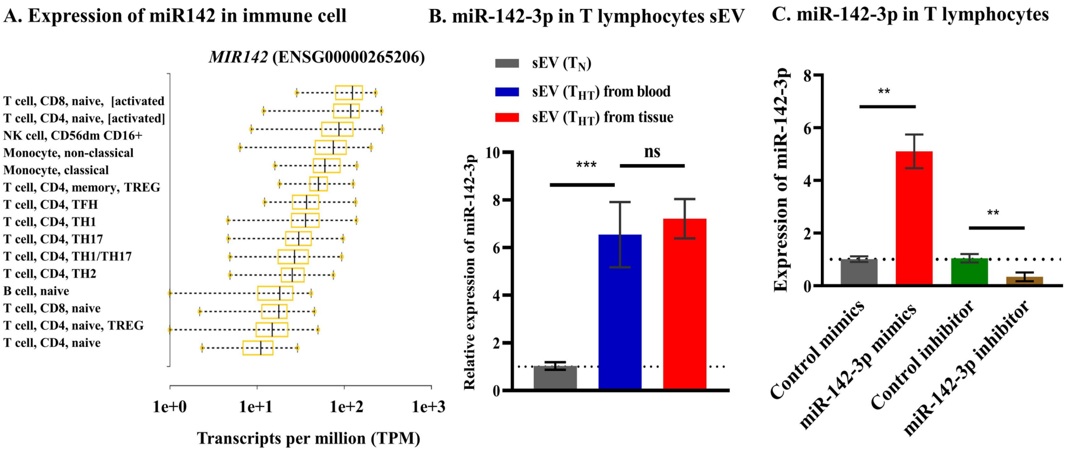

Figure S6. The miR-142-3p levels after transfection with mimics or inhibitor in thyrocyte cells, T lymphocyte migration and the effect of miR-142-3p on CXCL10 secretion

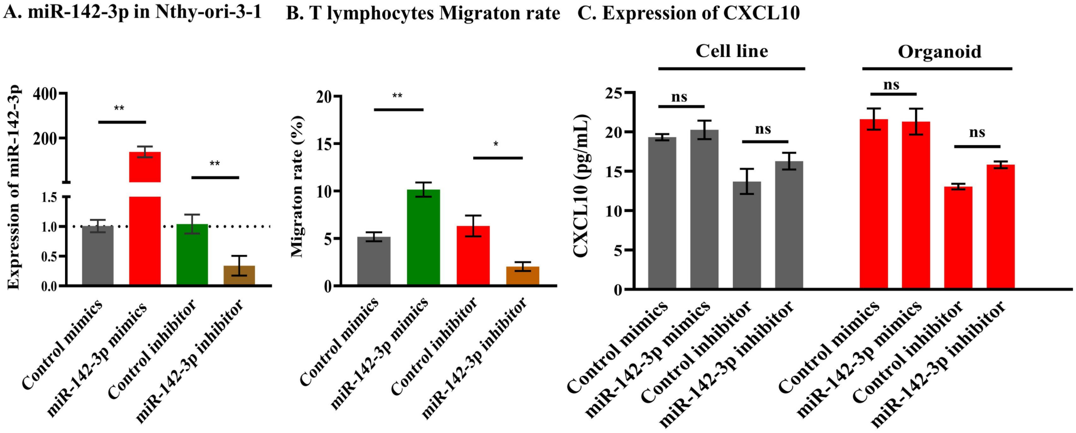

Figure S7. Predicted target and inhibition efficiency of siRNA for RAC1

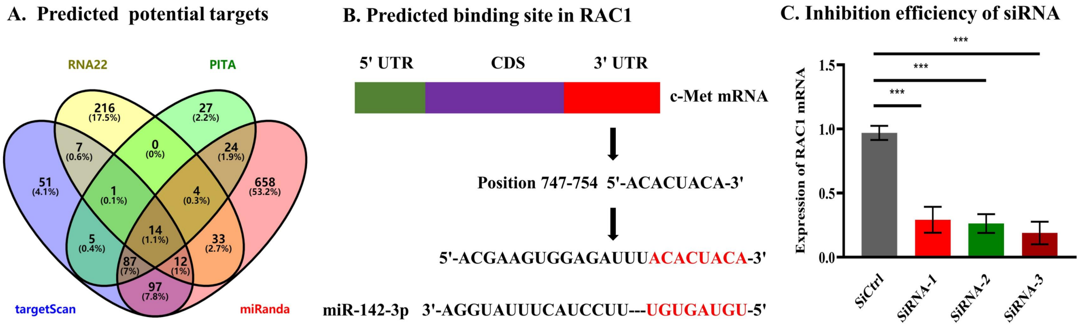

Supplement: Supplementary file 1 — Additional file 1: Table S1. The baseline demographics and clinical characteristics of enrolled subjects. Table S2. Univariate and multiple analysis for difference miRNAs. Fig S1. The uncropped original western blots from the main figures. Fig S2. The lowest RNA concentration of tissue sEV total RNA and levels of tissue sEV miR-103a-3p. Fig S3. Tissue sEVs isolated from HT patients promote apoptosis of thyrocyte cells, which can be prevented by a miR-142-3p inhibitor. Fig S4. Biological function enrichment analysis and validation of tissue sEV miRNAs. Fig S5. The levels of miR-142-3p in specific immune cell types and T lymphocyte-derived sEVs. Fig S6. The miR 142-3p levels after transfection with mimics or inhibitor in thyrocyte cells, T lymphocyte migration and the effect of miR-142-3p on CXCL10 secretion. Fig S7. Predicted target and inhibition efficiency of siRNA for RAC1. [file 12916_2023_2914_MOESM1_ESM.pdf]
