# Supplementary material for: miR-142-3p encapsulated in T lymphocyte-derived tissue small extracellular vesicles induces Treg function defect and thyrocyte destruction in Hashimoto’s thyroiditis
Source: BMC Med. 2023 Jun 6;21:206. doi: 10.1186/s12916-023-02914-7 (PMC10242775; doi:10.1186/s12916-023-02914-7)
Supplement: Supplementary file 3 — Additional file 3. The DEM expressed in all testing samples. [file 12916_2023_2914_MOESM3_ESM.pdf]

ID	N-01	N-02	N-03	N-04	N-05	N-06	N-07	N-08	N-09	N-10	HT-1	HT-2	HT-3	HT-4	HT-5	HT-6	HT-7	HT-8	HT-9	HT-10	PValue	FDR	log2FC	regulated
hsa-miR-155-5p	108.06036	139.82305	73.517484	125.99715	136.86374	2872.9222	637.38219	86.226719	63.83157	874.88339	695.92366	2298.1221	1010.3878	2669.762	1761.2512	4284.5559	667.80579	1119.0917	330.40855	2279.5132	0.0038862	0.4111802	3.4538692	up
hsa-miR-150-5p	877.9904	1019.744	323.4079	639.9905	620.9768	41399.623	6052.407	847.89607	889.6525	3136.1205	10284.098	5763.1033	4072.8463	26768.096	8324.5994	24869.053	9561.9343	7573.6302	3183.2043	9550.1354	0.0038862	0.4111802	3.3380231	up
hsa-miR-142-3p	224.22524	421.8799	97.678159	125.37186	100.66837	1908.2474	622.85496	57.484479	118.35437	1027.082	678.62169	1645.6707	935.50284	3076.0302	1488.3813	3928.4227	1753.4565	1181.4946	499.64219	2451.2384	0.0020892	0.3501717	3.164264	up
hsa-miR-146a-5p	1314.2841	1779.1278	1023.3772	1198.6925	984.0616	11374.119	3146.9611	1523.3387	1009.3367	4009.9685	6039.3485	5253.4706	3574.3585	8896.4431	7971.1089	14004.252	4281.0455	5100.3958	3439.4724	6290.1269	0.0020892	0.3501717	1.992651	up
hsa-miR-146b-5p	148.58299	306.16427	80.075381	97.85883	170.7969	862.17812	355.91712	459.87583	121.01402	1137.8661	473.88172	1932.6104	894.14847	986.65119	1370.5511	1331.3901	684.59421	488.8226	394.87851	1193.7669	0.0020892	0.3501717	1.9794025	up
hsa-miR-133a-3p	55.380933	16.875196	13.460948	10.317385	10.179948	33.160697	7.2636147	71.855599	29.256136	15.530474	38.448821	14.759379	24.589083	5839.0681	128.16617	43.831774	42.903724	35.361635	51.575968	52.625456	0.035463	0.5953116	1.4203763	up
hsa-miR-342-3p	1267.0077	1632.0725	1057.8924	1063.6286	978.40607	13659.192	3243.204	2069.4413	1787.284	2643.2867	4112.1014	3592.6064	2173.8985	10270.707	4760.753	9308.7729	4435.872	3240.7899	2736.7498	4686.4354	0.005196	0.4111802	1.3218576	up
hsa-miR-223-3p	688.88478	354.37911	232.28764	212.28801	268.07195	1486.2021	664.62075	359.27799	414.9052	772.38226	792.04571	633.78509	552.13668	1685.1837	1451.1718	1265.6425	554.01765	1002.6064	958.99066	944.48845	0.0146896	0.5953116	1.2978918	up
hsa-miR-374a-5p	105.35885	48.214845	34.515251	27.825674	20.359895	18.087653	14.527229	14.37112	18.617541	52.803613	26.914175	32.991552	53.648908	39.383136	72.35187	54.789717	39.172965	37.441731	54.799466	55.395217	0.0432571	0.5953116	0.9491266	up
hsa-miR-382-5p	12.15679	16.875196	32.789488	30.326858	27.146527	9.0438264	9.0795184	43.113359	49.203502	23.813394	26.914175	52.960124	21.236026	111.93102	53.747103	60.268689	24.249931	31.201443	48.35247	49.855695	0.0232306	0.5953116	0.9464799	up
hsa-miR-21-5p	2197.6775	1998.5053	1243.5845	1339.3841	1217.0693	3035.7111	1810.456	646.70039	807.20339	4343.356	1954.1613	5009.9409	2625.4434	2663.5437	4671.8636	9495.058	2742.1076	3155.5059	2029.192	6700.0515	0.005196	0.4111802	0.9048527	up
hsa-miR-193a-5p	85.097531	26.518165	63.162909	58.777828	105.19279	57.277567	108.95422	43.113359	106.38595	82.829197	71.130318	94.199564	136.35764	126.44059	134.36776	153.41121	83.942069	141.44654	58.022964	138.48804	0.0185434	0.5953116	0.8370997	up
hsa-miR-423-5p	456.55501	296.5213	367.58742	309.20889	434.34443	741.59376	483.03038	574.84479	742.042	364.44847	421.97581	621.6303	833.79345	1131.747	737.98907	1123.1892	669.67117	969.32482	472.24246	875.24443	0.0089307	0.542909	0.8190671	up
hsa-miR-92a-3p	729.40741	677.41858	419.70545	450.83844	469.40869	1633.918	935.1904	287.4224	1372.3788	534.24832	845.87406	638.56018	941.09126	1204.2948	950.91029	1840.9345	1128.5545	1329.1815	847.77998	1202.0762	0.0432571	0.5953116	0.7792191	up
hsa-let-7d-3p	183.70261	183.21641	104.58121	106.61297	234.13879	177.86192	292.36049	143.7112	280.59294	176.01204	276.83151	210.5382	255.95	165.82373	359.69215	323.25933	333.9029	320.33481	386.81976	296.36441	0.005196	0.4111802	0.7722552	up
hsa-let-7b-5p	40688.777	46336.877	49741.653	53020.102	43005.754	48001.616	61214.113	64741.895	85169.932	45043.553	53542.867	74181.072	76326.749	67005.223	80688.872	76333.034	67370.039	76329.129	55856.774	75293.179	0.005196	0.4111802	0.6128277	up
hsa-miR-130a-3p	815.8557	600.27482	391.74809	403.31594	561.02822	244.18331	350.46941	316.16464	243.35786	689.55306	619.02601	316.45844	296.18668	107.78542	159.17411	142.45326	264.88386	193.44895	349.74953	254.818	0.0232306	0.5953116	-0.613387	down
hsa-let-7a-3p	22.962826	81.965237	17.257625	12.505921	10.179948	15.073044	19.974941	86.226719	18.617541	27.954854	19.22441	9.5501862	6.7061135	14.509576	22.739159	5.4789717	9.3268965	14.560673	24.176235	8.3092826	0.035463	0.5953116	-0.681697	down
hsa-let-7f-2-3p	22.962826	72.322268	16.912473	10.942681	10.179948	15.073044	19.974941	86.226719	18.617541	26.919489	16.340749	9.1160869	6.7061135	14.509576	20.671963	5.4789717	9.3268965	14.560673	20.952737	8.3092826	0.0185434	0.5953116	-0.695149	down
hsa-miR-15b-5p	726.7059	641.25744	737.5909	700.64421	764.62717	955.63099	604.69593	287.4224	462.77888	750.6396	703.61342	614.25061	347.60022	679.87729	537.47103	427.35979	373.07586	359.85664	448.06622	387.76652	0.0232306	0.5953116	-0.705282	down
hsa-miR-29c-3p	26400.496	23885.634	17204.472	11849.047	15612.646	32307.562	11158.728	18337.549	6767.4762	20615.152	18489.077	9314.0362	8901.248	12244.01	10757.689	8267.7683	14796.189	5855.4708	17564.841	8445.0009	0.035463	0.5953116	-0.824361	down
hsa-miR-324-3p	14.858299	21.69668	23.47037	16.570345	22.622106	72.350611	39.949881	172.45344	35.905258	27.954854	29.797836	13.89118	14.529913	14.509576	16.53757	5.4789717	16.788414	12.480577	16.11749	13.848804	0.0015047	0.3501717	-0.82446	down
hsa-miR-29a-3p	25627.864	22943.034	16619.093	11278.465	14995.063	31436.341	10661.171	17618.993	6522.7885	19502.134	17754.704	8710.638	8478.7629	11570.351	10087.918	7314.4272	14327.978	5360.4079	16736.402	7871.6604	0.035463	0.5953116	-0.86498	down
hsa-miR-199b-5p	2635.322	5732.7451	1797.5542	1453.8133	1729.46	847.10507	1027.8015	4340.0782	855.07707	2106.9677	1081.3731	760.108	606.90327	945.19526	1418.0967	679.39249	1788.8988	728.03366	3529.7303	612.11715	0.0288056	0.5953116	-1.048419	down
hsa-miR-532-5p	471.41331	520.72033	248.16465	280.75792	256.7609	476.30819	368.62845	617.95815	337.77539	301.2912	400.82896	167.99646	97.238646	167.89653	146.77094	153.41121	294.72993	149.76693	460.96022	160.64613	0.0068415	0.4640462	-1.104412	down
hsa-miR-497-5p	624.04856	443.57658	347.56857	254.49549	476.19532	437.11828	350.46941	229.93792	106.38595	469.02033	341.23328	194.04242	127.41616	172.04212	146.77094	186.28504	193.99945	114.40529	296.56182	177.2647	0.005196	0.4111802	-1.115288	down
hsa-miR-424-5p	316.07654	318.21798	461.12375	276.0682	594.96138	147.71583	148.9041	244.30904	128.99296	565.30927	155.71772	118.07503	214.59563	68.402288	225.32439	120.53738	179.07641	108.165	149.89266	83.092826	0.0068415	0.4640462	-1.130694	down
hsa-miR-542-5p	179.65034	173.57344	64.198366	72.53434	98.40616	141.68661	103.50651	502.98919	106.38595	60.051168	137.45453	24.309565	79.355677	45.601526	138.50215	16.436915	55.961379	33.281539	46.740721	13.848804	0.0068415	0.4640462	-1.184588	down
hsa-miR-4324	29.716598	9.6429691	30.373421	14.694457	42.982001	39.189914	12.711326	186.82456	33.245609	32.096314	28.836616	11.720683	15.647598	12.43678	14.470374	5.4789717	22.384552	8.3203847	24.176235	11.079043	0.0114962	0.5953116	-1.215168	down
hsa-miR-574-5p	21.612071	21.69668	26.23159	19.071529	39.588685	30.146088	52.661207	114.96896	47.873677	57.980438	26.914175	22.573167	12.294541	31.091949	14.470374	5.4789717	11.192276	8.3203847	17.729239	11.079043	0.0028795	0.40211	-1.381535	down
hsa-miR-30c-1-3p	13.507545	55.447072	11.390033	14.381809	23.753211	21.102262	23.606748	71.855599	34.575434	17.601204	5.7673231	8.6819875	6.7061135	14.509576	12.403178	5.4789717	9.3268965	6.2402886	4.835247	13.848804	0.0003248	0.3501717	-1.53875	down
hsa-miR-1291	31.067353	38.571876	71.446569	533.37752	289.56295	201.97879	101.69061	488.61807	626.34728	46.591423	115.34646	130.22981	35.765939	31.091949	2.0671963	5.4789717	20.519172	91.524232	124.10467	11.079043	0.0288056	0.5953116	-2.183332	down
